# Editorial: Structural modeling and computational analyses of immune system molecules

**DOI:** 10.3389/fimmu.2023.1274670

**Published:** 2023-09-05

**Authors:** Dinler A. Antunes, Clara T. Schoeder, Minkyung Baek, Eduardo A. Donadi

**Affiliations:** ^1^ Department of Biology and Biochemistry, University of Houston, Houston, TX, United States; ^2^ Institute for Drug Discovery, Faculty of Medicine, Leipzig University, Leipzig, Germany; ^3^ Department of Biochemistry, University of Washington, Seattle, WA, United States; ^4^ Department of Medicine, Division of Clinical Immunology, Ribeirão Preto Medical School, Ribeirão Preto, SP, Brazil

**Keywords:** structural modeling and analysis, structural bioinformatics tools, immune system molecules, molecular docking, molecular dynamics, MHC, TCR - T cell receptor, BCR - B cell receptor

Immune system genes have evolved to cope with harmful environmental agents and to control self-injury, tasks that have been primarily performed by the T and B cell lymphocytes. In medullary thymus epithelial cells, the expression of the autoimmune regulator (AIRE) gene during the negative selection permits the expression of thousands of tissue-specific genes, allowing the elimination of selfreactive T cells (negative selection), the survival of non-self-reactive T cells (positive selection), and the generation of T regulatory cells (Santos et al.). These essential processes in T cell development are possible through the specific interactions between T cell receptors (TCRs) and major histocompatibility complex (MHC) receptors at the surface of thymocytes (1). Notably, infectious, autoimmune and cancer disorders have been associated with particular MHC class I or class II molecules, due to the differential peptide presentation capacity of different MHC allotypes. Considering the enormous polymorphism of MHC molecules in worldwide populations and the even greater diversity of peptides to be presented by these molecules, the study of the peptide-MHC binding has direct impacts on disease association, vaccine development, and transplantation. To handle the enormous diversity of potential antigens, somatic rearrangement of genes encoding both TCRs and B cell receptors (BCRs, or antibodies) permits these immune system cells to specifically recognize antigens, and perform effector functions against environmental agents (2). In turn, the specificity of these interactions is driven by structural features of these immune system molecules, and the structural characterization of these molecular complexes will be the key for several biomedical applications. However, experimental methods for protein structure determination cannot be deployed at the scale needed to study this diversity. Therefore, there is a constant need for reliable computational approaches to enable structural modeling and analysis of immune system molecules and macromolecular complexes. In this context, computational methods for modeling the 3D structure and dynamics of protein/protein and protein/peptide interactions (1–5), together with deep-learning algorithms (6, 7), have been developed and deployed to study how such diversity can influence immune responses. In this Research Topic, we present new computational strategies to broaden the understanding of these critical immune system molecules.

For instance, Santos et al. leveraged the analysis of protein/protein and protein/peptide interactions to understand the pathogenesis of the autoimmune polyendocrinopathy candidiasis ectodermal dystrophy (APECED), which is primarily caused by the AIRE SAND domain G228W mutation (**Figure 1**). They studied the interaction of the whole SAND domain with the SIRT-1 molecule, an important component of the AIRE complex. The results from structural modeling, molecular docking, and molecular dynamics of both the AIRE wild type G228 (glycine) and the AIRE mutant W228 (tryptophan) showed that this mutation negatively influenced the AIRE-SRT-1 interaction. The mutation impairs the downstream activation of the RNA polymerase II, which is responsible for the promiscuous tissue expression required for the negative selection in the thymus. To validate the *in silico* results, the authors performed an elegant *in vitro* study using surface plasmon resonance, coupling the 211-230 residues of the SAND domain with the SIRT-1 protein, showing similar results. Noteworthy, the validation of *in silico* studies, whenever possible, should be provided by a complementary strategy (e.g., *in vitro*, *in vivo*, and functional studies), as reported here.

**Figure 1:**
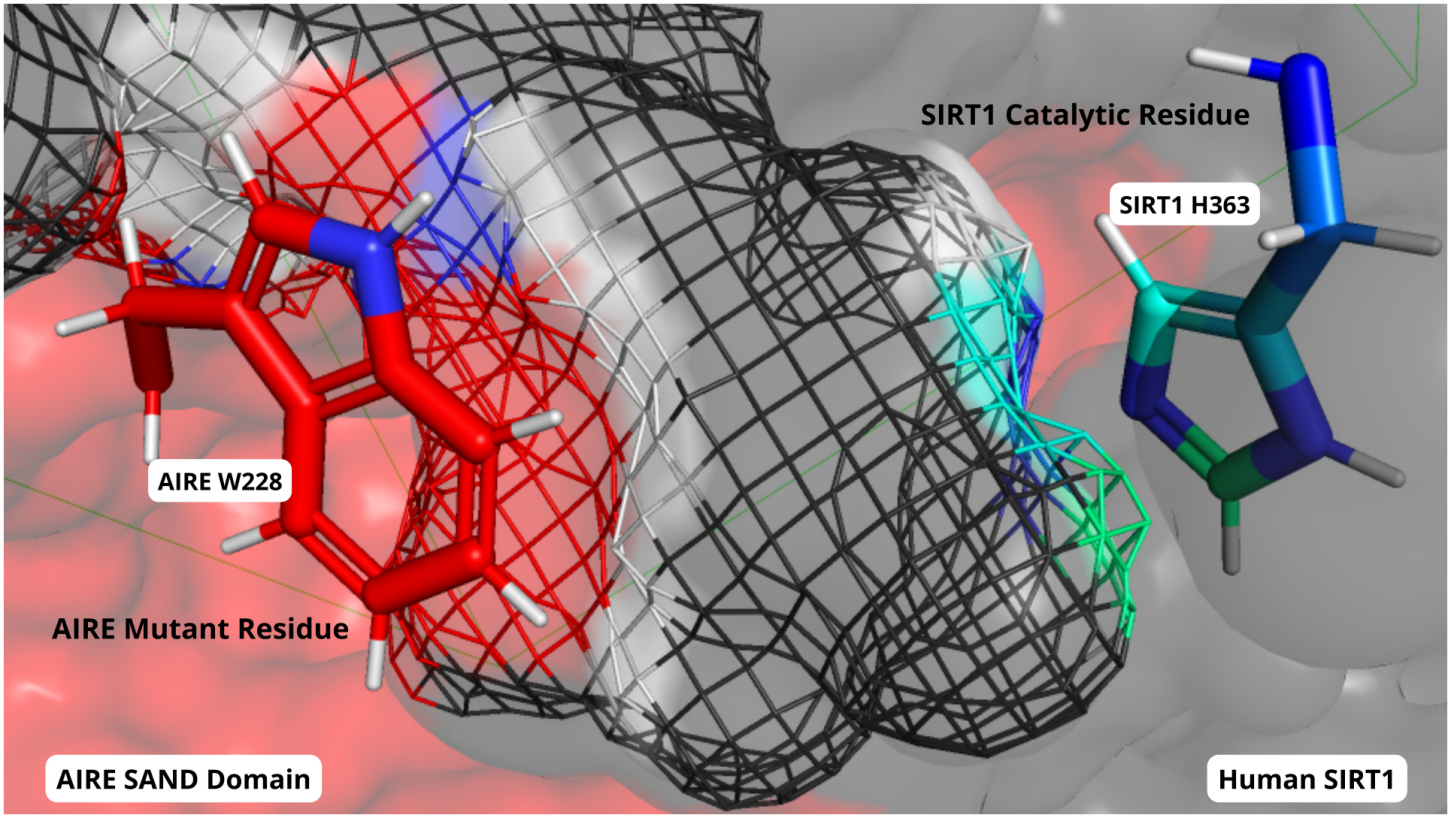
Close-up view of the molecular interactions between the mutant AIRE SAND domain (surface in red) and SIRT1 protein (depicted in dark grey) after 1 microsecond of molecular dynamics simulation. The mutated W228 residue within the AIRE SAND domain is prominently featured (side chain colored in red/blue), showing its proximity to the SIRT1’s catalytic H363 residue (side chain colored in blue/green). The relative positions of the W228 and H363 residues underscore the altered interaction dynamics between the mutant AIRE and SIRT1, as compared to the wild-type complex.

Also focused on protein/peptide interactions, Marzella et al. developed the PANDORA generic pipeline to model the 3D structures of peptides displayed by both class I and class II MHC molecules. Based on experimentally-determined structural templates from an extensive reference database, this friendly and freely-available algorithm uses anchor restraints to enable fast and accurate modeling of MHC-bound peptides. In parallel, Ochoa et al. developed the PanMHC-PARCE protocol to engineer peptides that can simultaneously bind to several class II MHC molecules, envisaging applications for the development of vaccines and immunotherapies with broader population coverage. The authors used this approach to improve the binding affinity of a *Plasmodium vivax* epitope towards multiple MHC molecules, and confirmed the predicted enhanced binding capacity experimentally. Additionally, the authors validated their engineered peptides by immunizing mice and observing interferon-*γ* production *in vivo*.

Going beyond epitopes recognized by T cell receptors in the context of the MHC molecules, the interactions between B cell receptors (antibodies) and their targets were studied by Tran et al. These large protein/protein complexes pose several computational challenges for modeling, regarding their size, the flexibility of the interacting surfaces, and the low resolution of available experimental data. To overcome these challenges, the authors revisited the hydrogen-deuterium exchange mass spectrometry (HDX-MS) methodology, which has been used for epitope-mapping of antibodies (8). The authors addressed the HDX-MS limitations, especially the peptide resolution, proposing that computational simulation of HDX-MS-generated data combined with protein docking will be able to overcome these limitations. Although the determination of antibody specificity is far from exhibiting a complete resolution, the computational strategy used by the authors represents an additional step to solve the interpretation of this complex protein/protein interaction. Rather than studying these large antigen/antibody complexes, Cohen et al. focused on single heavy chain camelid antibodies (nanobody), as a cost-effective highly stable substitute for full-length antibodies. Considering that the nanobody domain presents long CDR3 loops, lacks the light chains, and can be administered by aerosolization, nanobodies may define antibody specificity and may be used as a therapeutic agent.

Taken together, this series encompass both specific contributions, as well as broader insights into the future of computational modeling for immunological applications. The specific contributions include i) the application of bioinformatics approaches to understand the diversity of the immune system molecules (Marzella et al., Ochoa et al., Tran et al., Cohen et al.), ii) the understanding of the disease pathogenesis (Santos et al.), iii) improvement of tools to validate *in silico* studies (Santos et al., Ochoa et al.), iii) new tools for pMHC modeling (PANDORA) and engineering (PanMHC-PARCE) (Marzella et al., Ochoa et al.), and the use of nanobodies to discriminate T/B cell epitopes (Cohen et al.). More broadly, these studies demonstrate the strength of integrative approaches combining experimental methods with computational approaches, to complement each other when dealing with complex systems such as those involved in immune responses (9). Further advances in this interface may improve vaccine development (10), predict disease pathogenesis, susceptibility and morbidity, and provide strategies to ameliorate disease treatment.

## Author contributions

DA: Conceptualization, Supervision, Writing – original draft, Writing – review & editing. CS: Data curation, Supervision, Writing – review & editing. MB: Data curation, Supervision, Writing – review & editing. ED: Data curation, Supervision, Writing – original draft, Writing – review & editing.
